# AMID: Accurate Magnetic Indoor Localization Using Deep Learning

**DOI:** 10.3390/s18051598

**Published:** 2018-05-17

**Authors:** Namkyoung Lee, Sumin Ahn, Dongsoo Han

**Affiliations:** School of Computing, Korea Institute of Science and Technology, Daejeon 34141, Korea; nlee@kaist.ac.kr (N.L.); smahn@kaist.ac.kr (S.A.)

**Keywords:** magnetic landmark, deep learning, recurrence plot

## Abstract

Geomagnetic-based indoor positioning has drawn a great attention from academia and industry due to its advantage of being operable without infrastructure support and its reliable signal characteristics. However, it must overcome the problems of ambiguity that originate with the nature of geomagnetic data. Most studies manage this problem by incorporating particle filters along with inertial sensors. However, they cannot yield reliable positioning results because the inertial sensors in smartphones cannot precisely predict the movement of users. There have been attempts to recognize the magnetic sequence pattern, but these attempts are proven only in a one-dimensional space, because magnetic intensity fluctuates severely with even a slight change of locations. This paper proposes accurate magnetic indoor localization using deep learning (AMID), an indoor positioning system that recognizes magnetic sequence patterns using a deep neural network. Features are extracted from magnetic sequences, and then the deep neural network is used for classifying the sequences by patterns that are generated by nearby magnetic landmarks. Locations are estimated by detecting the landmarks. AMID manifested the proposed features and deep learning as an outstanding classifier, revealing the potential of accurate magnetic positioning with smartphone sensors alone. The landmark detection accuracy was over 80% in a two-dimensional environment.

## 1. Introduction

Indoor positioning has been studied for many years owing the increasing demands on indoor positioning services. Indoor navigation, indoor location-based safety management, social media, advertisement and entertainment are some examples. Wi-Fi signals or geomagnetic data are typically used for indoor positioning services. Each method has advantages and disadvantages for positioning in terms of accuracy, effort, and cost. In practice, Wi-Fi is one of the most commonly used wireless signals for indoor positioning. Because Wi-Fi access points (AP)s have already been installed in many buildings, the deployment cost is close to zero. However, Wi-Fi signals are not stable enough to yield reliable positioning results owing to the multi-path problem, incorrect floor detection problem, and so on [1,2].

Geomagnetic-based positioning methods have been actively studied recently. The methods utilize distorted indoor magnetic fields caused by ferromagnetic objects, such as steel frames and electrical appliances. Geomagnetic data can be obtained anywhere; thus, no infrastructure is required. Moreover, magnetic signals are relatively stable. However, the available data set scanned from smartphones is a collection of vectors with only three values in a Cartesian space. Moreover, there is a great deal of ambiguity in geomagnetic data, especially in a large area [3,4]. Consequently, it is challenging to build a standalone magnetic positioning system.

Some studies on positioning methods with low-quality sensors, including those in smartphones, borrowed techniques developed for high-quality sensors. Haverinen and Kemppainen [5] applied simultaneous localization and mapping(SLAM), which is usually used by robots, for a person with attached sensors. However, the positioning performance was not as accurate as that of robots because the inertial sensors attached to the person indirectly shows the movement of the person. Using particle filters with smartphones is one approach used to cope with the ambiguity issue. Others attempted to improve positioning accuracy by reorganizing magnetic data that are not affected by the users’ orientation, or by using sophisticated pedestrian dead-reckonings to infer human motions more precisely [3,6]. Furthermore, some studies made use of floor plans and the patterns of human locomotion to converge particles faster [7,8]. Other studies improved indoor positioning accuracy by fusing radio signals and geomagnetic data together [9,10].

One possible approach estimating locations is analyzing the patterns of magnetic sequences [11,12,13]. The feasibility of using pattern recognition techniques has been proven in a one-dimensional space. However, there are several drawbacks. First, a simple sequence matching algorithm such as dynamic time warping (DTW) cannot effectively handle the data obtained from magnetometers. Even along the same path, the sequences show variations because the magnetic intensity on the path fluctuates with a slight change in location. There are studies that extract features from magnetic sequences to address this problem, but they cannot provide enough information for two-dimensional positioning [13,14]. Second, there are limitations in scalability because there are too many possible paths in a two-dimensional space. LocateMe [12] reduced the search space by inferring locations from the positions of nearby ferromagnetic structures. It is known that paths affected by the same magnetic landmarks have similar patterns, but these are not evaluated in two-dimensional space. There is as yet no standalone magnetic positioning system operating in a 2D space with high accuracy.

In this paper, we propose accurate magnetic indoor localization using Deep Learning (AMID), an indoor positioning system that recognizes magnetic sequence patterns using a deep neural network(DNN). In order to identify the patterns, AMID extracts new types of features, including recurrence plots(RPs) from the sequences. The features are informative enough for two-dimensional positioning. They can be regarded as a generalization of a path with slight variations, because they represent the shape of curves. Moreover, AMID can estimate locations in a two-dimensional space by taking an approach similar to that of LocateMe [12]. This method has become more practical by using a new way to locate magnetic landmarks automatically. In addition, a deep learning-based classifier is used to enhance the accuracy of magnetic landmark detection. Deep learning, which has proven powerful for image processing using the feature fusion of mel-frequency cepstral coefficients(MFCCs) and linear frequency cepstral coefficients(LFCCs) for voice recognition, greatly contributes to the classification of image features, e.g., RPs, without additional processing.

The rest of this paper is organized as follows. Section 2 introduces related research on magnetic positioning methods. Section 3 presents preliminaries for a better understanding of the proposed method. Section 4 presents the system design of AMID. The following two sections describe the methodology in three phases. Section 7 presents an evaluation of the proposed system. Finally, Section 8 concludes this paper.

## 2. Preliminaries

**Figure 1:**
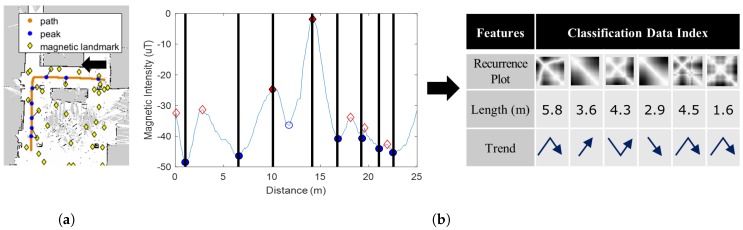
Intuition of magnetic localization with deep learning. (**a**): Magnetic landmarks (**b**): Feature extraction.

In this section, we briefly explain the positioning process of AMID and the feasibility of a deep learning-based magnetic positioning method. Figure 1 depicts the intuition of magnetic localization in AMID. AMID utilizes the magnetic sequence patterns observed when a person walks from one magnetic landmark to another. The left image map of Figure 1a represents magnetic landmarks. The landmarks are detected by peaks on a magnetic grid map, and their location can be estimated by finding the locations of peaks in a magnetic graph. The magnetic graph in Figure 1b shows the positions of the magnetic landmarks as filled markers. These markers on the graph represent peaks exceeding a particular threshold. In other words, a magnetic sequence can be represented with magnetic landmarks. As shown in the graph, magnetic sequences have unique patterns. By analyzing these patterns, AMID estimates the magnetic landmarks in close proximity to user.

The process of our magnetic localization is similar to that of the speech recognition. It is well known that deep learning-based speech recognition outperforms other existing speech recognition techniques. The speech recognition method consists of two phases: feature extraction and DNN training. In the feature extraction phase, slides of audio signals in the time domain are converted to MFCCs and LFCCs. The cepstra of the MFCC and LFCC are fused and used at the DNN training phase as image features. A convolutional neural network is used because the inputs are images. Training data are labeled with the correct phonemes. A trained model can predict phonemes for given audio signals. The magnetic localization model follows the same process. A sequence of magnetic data is divided into training data depending on reference points, which are peaks in the graph of Figure 1b. Features are then extracted, such as image features and RPs. In addition to the DNN structure of speech recognition, auxiliary features other than RPs are inserted at the first fully connected layer. Finally, given the magnetic sequences, the DNN predicts magnetic landmarks that match to the sequences.

## 3. AMID System Design

**Figure 2:**
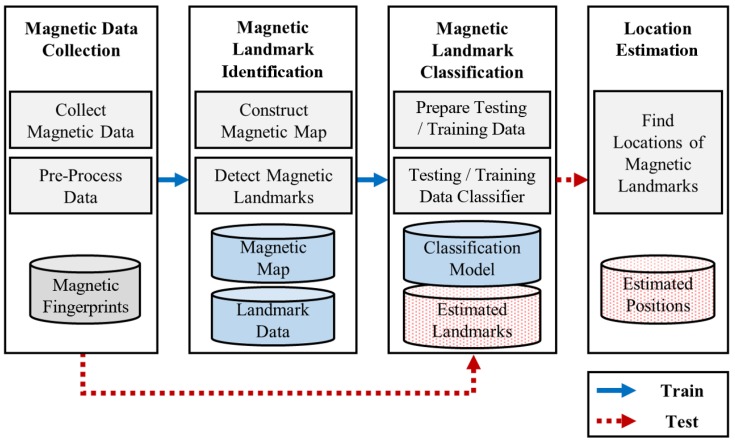
Schematic view of AMID.

Figure 2 shows a schematic view of the AMID localization process. Magnetic data collection, magnetic landmark localization, magnetic landmark classification, and localization prediction are the main steps of the process. First, in the collection step, magnetic data for training are gathered by maneuvering a mobile robot. When magnetic data are collected by a sensor in a smartphone mounted on a robot, the location of the robot is collected as well. The magnetic fingerprints are constructed by matching the magnetic sensor data and with the corresponding locations. In addition, data measured by inertial sensors are collected simultaneously. The data are used to estimate user walking distances to support the classification step. Pre-processing is required to remove the noise of the magnetometer and reorganize magnetic elements for improved positioning performance. Second, in the magnetic landmark identification step, magnetic landmark data and geomagnetic statistics are generated. A magnetic map is required to find magnetic landmarks. The map can be constructed by interpolating the magnetic fingerprints collected by the robot. The peaks in the map are the landmark candidates. By finding these points, the locations of magnetic landmarks can be identified. The locations are stored and used for data labeling in the classification step. Third, in the classification step, a classification model is trained with the data from the collection step. Magnetic sequences, which are divided by reference points in the magnetic data, are labeled with corresponding magnetic landmarks. The magnetic sequences are then used as input for the model. Finally, in the location estimation step, magnetic landmarks are detected on the constructed classification model. The location is estimated based on the detected landmarks. The following is a detailed description of the steps.

## 4. Magnetic Data Collection

The magnetic data collection step consists of data collection, noise reduction, magnetic data correction, and magnetic elements conversion. When magnetic fingerprints are generated, noise reduction and magnetic data correction procedures are performed to correct noise from the magnetic sensors and location-labeling errors of the data. In addition, the magnetometer’s three-axis data vector is converted to three magnetic elements.

### 4.1. Data Collection and Noise Reduction

Deep learning usually requires a large amount of training data. To obtain accurate magnetic data in a short time, robotic rather than manual data collection is used. The robot provides position-related data: (t,x,y,yaw,pitch,androll), which capture the time, x-coordinate, y-coordinate, and orientations in three planes. The robot utilizes SLAM to localize itself. Further, data from the magnetic sensor that consist of time (t) and the three-axis magnetic vector $\left(m_{x},m_{y},m_{z}\right)$ are obtained by a smartphone mounted on the robot. The magnetic data structure $D_{train}=\left(t,x,y,yaw,pitch,roll,m_{x},m_{y},m_{z}\right)$ used in the training is constructed by integrating these two sets of data by referencing their timestamps.

In the prediction phase magnetic data, $D_{prediction}=\left(t,Acc_{x},Acc_{y},Acc_{z},m_{x},m_{y},m_{z}\right)$, are assumed to be collected by users’ smartphones. In this case, instead of the position information (x,y,yaw,pitch,roll) obtained by the robot, the three-axis accelerometer data $\left(Acc_{x},Acc_{y},Acc_{z}\right)$ are gathered. The data from inertial sensor are used to estimate the step distance of users in the classification step. However, low-quality MEMS magnetometers in smartphones transmit a lot of noise. The magnetic sensor (BMM150) of the Nexus 6P we used typically generates noise up to 1.4 μT [15]. To remove the noise, we use a smoothing filer (moving average) with a particular window size.

### 4.2. Magnetic Data Correction

SLAM used by a robot may have positioning errors caused by false readings of the laser sensor (LIDAR). When the robot moves around to build a map, readings of the sensor may be distorted by reflective materials like glass walls, resulting in the construction of a distorted SLAM map. Magnetic data correction mitigates this problem by transforming the distorted SLAM map. A piecewise-linear transformation, which compares the location of points of interest on both maps, was used to reduce the position errors in the magnetic data.

### 4.3. Magnetic Data Elements Conversion

**Figure 3:**
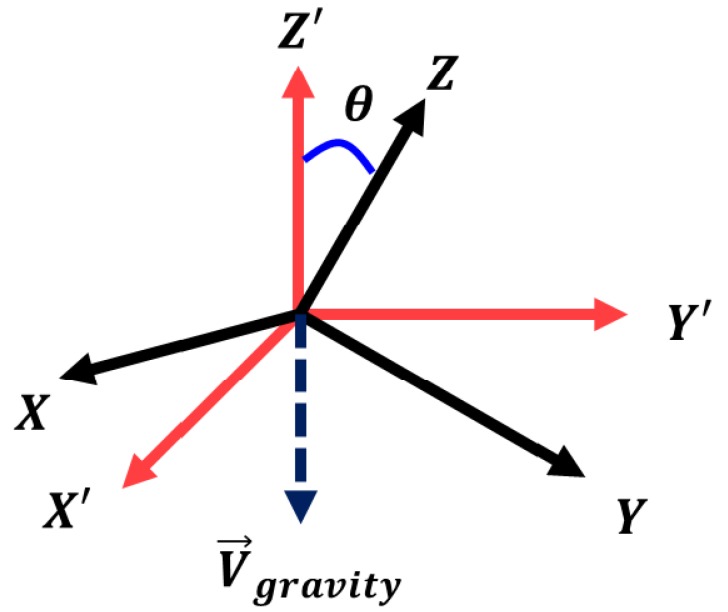
Magnetometer coordinates and transformed coordinates.

Heading estimation using smartphones is error-prone because a gyroscope is rarely free from cumulative integration errors. AMID uses three magnetic elements $\left(m_{z}^{\prime },m_{xy}^{\prime },m_{xyz}^{\prime }\right)$ that are not affected by orientation changes, where the *Z* coordinate of the magnetometer is transformed by using $Z^{\prime }$, which is opposite to the direction of gravity, as depicted in Figure 3. These elements can be obtained by calculating the angle difference θ between $Z^{\prime }$ and *Z* as follows:

(1) $$\begin{array}{c} \begin{array}{cc} m_{z}^{\prime } & =m_{z}\cdot \cos\theta +m_{xy}\cdot \sqrt[{2}]{1-\cos^{2}\theta }\\ m_{xyz}^{\prime } & =\sqrt[{2}]{{m_{x}}^{2}+{m_{y}}^{2}+{m_{z}}^{2}}\\ m_{xy}^{\prime } & =\sqrt[{2}]{{m_{xyz}^{\prime }}^{2}-{m_{z}^{\prime }}^{2}} \end{array} \end{array}$$

where $\;\cos\theta =\frac{\bar{Z}\cdot \bar{Z^{\prime }}}{|\bar{Z}\left|\cdot \right|\bar{Z^{\prime }}|},\;m_{xy}=\sqrt[{2}]{{m_{x}}^{2}+{m_{y}}^{2}}$

In Equation (1), *Z* represents a unit vector of the *Z* axis (0,0,1), and $Z^{\prime }$ is opposite to the gravitational vector that can be obtained through an API in the smartphone operating system [16,17].

## 5. Magnetic Landmark Identification

AMID constructs magnetic maps, and detects magnetic landmarks to automatically identify magnetic landmarks. A magnetic grid map is constructed by interpolating magnetic data. Then, the smoothing process corrects the error caused by the sensor readings offset.

**Figure 4:**
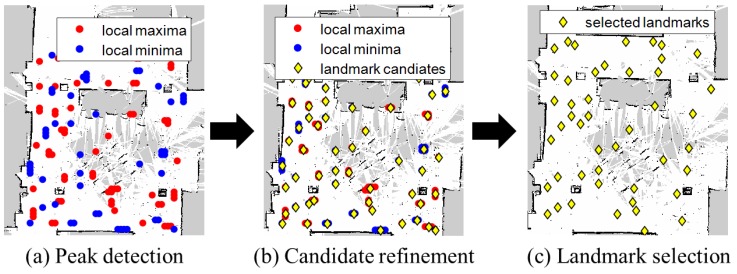
The process of magnetic landmark identification.

Landmark detection consists of local minima/maxima (peaks) detection, magnetic landmark candidate refinement, and magnetic landmark selection as shown in Figure 4. By finding the peaks on the map, magnetic landmark candidates are chosen. Then magnetic landmark candidate refinement removes outliers that are caused by low-quality smartphone sensors and the indoor environment. Finally, magnetic landmark selection finds landmarks by analyzing magnetic sequence patterns.

### 5.1. Magnetic Map Construction

Constructing a high-resolution magnetic map is essential for the localization process because the quality of landmark identification depends on the quality of the map. Because the high-resolution map with dense magnetic data represents the actual indoor magnetic environment precisely, magnetic landmarks can be identified accurately. The large amount of data collected by the robot makes it possible to construct a high-resolution map. By interpolating these data, a refined magnetic grid map is constructed. The magnetic map structure $\left(x,y,m_{z}^{\prime },m_{xy}^{\prime },m_{xyz}^{\prime }\right)$ has position (x,y) at particular intervals, and converts three magnetic elements $\left(m_{z}^{\prime },m_{xy}^{\prime },m_{xyz}^{\prime }\right)$ at the position.

While the robot freely collects magnetic data, paths can overlap each other. The interpolation process takes an average if there are several geomagnetic values at the same location. In the case of data vacancy at a certain area, linear interpolation is performed at intervals of 0.3 m. We have observed that a grid resolution of 0.3 m or less is required to generate an accurate magnetic map. If there is a gap exceeding 0.6 m in the magnetic data, the robot collects magnetic data for the gap.

One problem with the smartphone’s magnetic sensor in constructing the magnetic map is that the sensor needs to be calibrated continuously. If it isn’t calibrated, the sensor values may become biased toward a particular axis. Because the robot usually collects magnetic data for 2 h without calibration, some magnetic data may be biased. The biased data can be distinguished by checking offset occurrence between the current data and the previous data. We have observed that the offset affects the data less than 1 μT, which is not a large number. However, this offset still generates noises on the magnetic map. Therefore, to reduce the noise, a two-dimensional smoothing filter is applied to the map as defined below:

(2) $$m^{\prime }=\left(m*f\right),\;where\;f=\frac{1}{8\cdot a+b}\cdot \left[\begin{array}{ccc} a & a & a\\ a & b & a\\ a & a & a \end{array}\right]$$

### 5.2. Magnetic Landmark Identification

We explain three processes for magnetic landmark detection.

- **Local Minima/Maxima Detection:** Magnetic landmarks are ferromagnetic objects that have larger or smaller magnetic intensities than their surroundings. Therefore, magnetic landmark candidates can be identified by finding the local minima/maxima in a magnetic map. In this paper, we define local minima/maxima as values of the location on the map that are less or greater than the values of their 8-connected neighborhoods.
    
- **Magnetic Landmark Candidate Refinement:** Not all points can be used as magnetic landmarks for positioning. Outliers exist among these points depending on the indoor geomagnetic environment and magnetic landmark characteristics. In some areas, magnetic intensity rarely changes. Further, the magnetic intensities of some magnetic landmarks fluctuate over time. Fluctuation is usually observed due to AC electric motors. These circumstances generate clustered local minima/maxima. Magnetic landmark candidate refinement manages this problem. We use a Euclidean distance-based hierarchical tree to group these points as one magnetic landmark candidate.
    
- **Magnetic Landmark Selection:** Most of the landmark candidates have much higher or lower values than the mean magnetic intensity. However, the magnetic intensity of some candidates is similar to the average intensities, so no magnetic sequence pattern is created. To filter out these candidates, we apply particular thresholds to select the final magnetic landmark to be used for localization as follows:
  (3) $$peak_{min}\leq \mu -0.5\cdot \sigma \;peak_{max}\geq \mu +0.5\cdot \sigma$$
  where $peak_{min}$ and $peak_{max}$ denote local minima and local maxima. In addition, μ and σ represent the mean and the standard deviation of magnetic map values in Equation (3). The upper and lower limits can be set differently depending on the indoor environment, but that setting is generally effective for many cases. The selected landmarks are given unique indexes idx and stored as landmark data $\left(idx_{i},x_{i},y_{i}\right)$ along with the landmark locations $\left(x_{i},y_{i}\right)$.

## 6. Classification Model Training and Location Estimation

This step consists of data preparation, feature extraction, classifier training, and location estimation. Data preparation is performed to provide inputs for each phase. Both phases undergo domain conversion depending on the type of magnetic data received. The magnetic data separation allows magnetic sequences that match magnetic landmark to be extracted from the data. Additionally, in the training phase, location-labeling of training data for supervised learning is performed. After the feature extraction, which determines the features for the DNN classifier, the DNN is trained and tested to estimate locations.

In Equation (2), *m* and $m^{\prime }$ denote the magnetic map before and after the smoothing, and, *f* denotes the smoothing filter. Parameters *a* and *b* determine the degree of smoothing.

### 6.1. Training/Test Data Preparation

The data preparation step consists of domain conversion, magnetic data separation, and training data labeling as shown in Algorithm 1.

- **Domain Conversion (Line 1–6):** Because the features to be used for the DNN are extracted from the distance domain, it is necessary to convert the time domain magnetic data to the distance domain data. The conversion methods differ for each phase because the magnetic data structure depends on each phase. In the training phase, cumulative Euclidean distance is obtained from the distances between the magnetic data. Moreover, distance is estimated by step length estimation in the test phase. For the simplicity, we use Weinberg’s method [18], which uses accelerometer data to estimate users’ strides as shown below:
  (4) $$L=k\cdot \sqrt[{4}]{∥peak_{max}-peak_{min}∥}$$
  where *k* is a user-dependent parameter that varies depending on the user and the position of the smartphone. Therefore, this parameter requires a classifier to identify users and positions. $Peak_{min}$ and $Peak_{max}$ denote the minimum and maximum peak values given a particular window size. We set a default window size of 1 m. After the first estimation, we set the window size to 1.5 times that of the previous estimation.
    
- **Magnetic Data Separation (Line 8–14):** A magnetic landmark classification problem requires classification data that infer patterns using magnetic landmarks. Magnetic data separation divides the magnetic data in the distance domain into classification data using peaks in the magnetic data as the reference points. The occurrence of peaks implies that magnetic landmarks may be located close to one another. As in the case of the magnetic landmark selection, not all peaks can be reference points. Some local minima have higher magnetic values than the mean of the magnetic map. Likewise, some maxima have lower magnetic values than the average. These peaks usually appear when a person passes between magnetic landmarks with minimum or maximum values. To manage this problem, we set the same threshold to the peaks as in the localization step to select the final reference points.
    
- **Training Data Labeling (Line 14–21):** Because classification is supervised learning, classification data in the training phase have a target. The target is a magnetic landmark that corresponds to of a magnetic sequence pattern. The target can be obtained by finding the nearest magnetic landmark at the location of the reference points. Because all landmark locations are provided from the localization step, we calculate Euclidean distances from the reference points to all landmarks. The landmark with the minimum distance is then selected. We use the index of the nearest landmark as the target.

**Algorithm 1** Classification Data Generation.
*M* : set of magnetic data
μ : mean of magnetic map values
σ : standard deviation of magnetic map values
*C* : Classfication data 1.5
1:**if** isTrainingPhase() **then**2:    *D* = set of Euclidean distance between adjacent magnetic data3:
**else**
4:    D= set of Estimated step lengths for each magnetic data5:
**end if**
6:$M^{\prime }$ = Convert_TimeToDistance(*M*, *D*)7: 8:Find a set of peaks *P*9:**for** each peak ∈P **do**10:    **if** |p-μ|≥0.5·σ **then**11:        Set peak *p* as reference point $p_{ref}$12:    **end if**13:
**end for**
14:C = Divide each piece of magnetic data in *M*’ using each reference point as a boundary $P_{ref}$15: 16:**if** isTrainingPhase() **then**17:    **for** *p* in $P_{ref}$ **do**18:        Find index idx of nearest magnetic landmark from p19:        Label classification data *C* with idx20:    **end for**21:
**end if**
22:Return classification data *C*


### 6.2. Feature Extraction

AMID uses four features: RP, trend, sequence length, and peak values. The recurrence plot is the key features to categorize the sequence patterns. Other features are used as auxiliary inputs.

**Figure 5:**
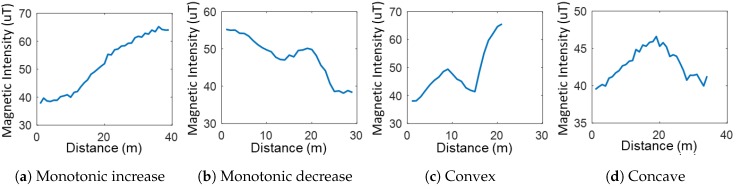

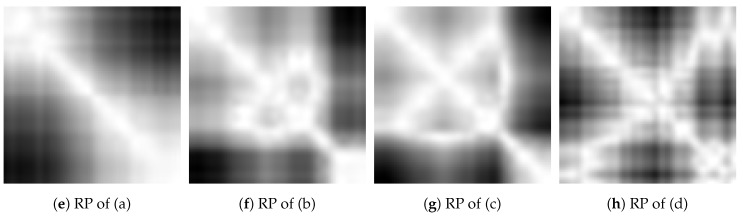
Examples of recurrence plot.

- **RP:** RP is usually used to analyze shapes or patterns in time series data [19]. We use the RPs to identify shapes in a magnetic sequence. This is the first case where an RP is used as an image feature to estimate locations. The image feature can be obtained by applying the RP as follows:
  (5) $$\begin{array}{c} R\left(i,j\right)=1-\frac{∥\vec{m_{i}}-\vec{m_{j}}∥}{∥\vec{m_{k}}-\vec{m_{l}}∥},\;\vec{m_{i}},\vec{m_{j}}\in R^{m}\\ \;\left(k,l\right)=\max_{argi,j}∥\vec{m_{i}}-\vec{m_{j}}∥,\;i,j=1,...,N \end{array}$$
  where *m* denotes the magnetic data element in the distance domain. Because some sequences have extremely long data length, we set the maximum sequence length to seven meters which covers over 90% of sequences. *R* is a normalized Euclidean distance matrix that finds all distances among all components. The RP is free from offsets of the sensor readings among smartphones because the RP only calculates gradient among the data elements.
  The RP generates unique images depending on the shape of curves as shown in Figure 5. The four images in Figure 5e–h are generated from the magnetic sequences in Figure 5a–d. For example, the shape of a straight line as shown in Figure 5a is the case where the trend is monotonic increase or decrease. Figure 5h shows the case where the shape of the magnetic sequence has convex or concave curve. Finally, other images have complex curve shapes in which the magnetic data have one or more fluctuations.
- **Auxiliary Inputs:** Although the RP captures many features in magnetic sequences, there is no distinction in terms of the sequence’s direction. To solve this problem, additional features are extracted in addition to the RP: trend, sequence length, and peak values. We use the types of peaks to sort the trend. For convenience, local minima and local maxima are labeled with 0 and 1 in order. Because magnetic sequences encompass two reference points as peaks, four combinations are possible: monotonic increase (01), monotonic decrease (10), convex (11), and concave (00). Furthermore, sequence length and values of reference points can be used as features. These two features help to classify the monotonic shape of magnetic sequences. For example, the RP cannot segregate magnetic sequences with different targets, whose trends show a steady increase or decrease. In such a case, sequence length and peak values.

### 6.3. Classifier Training and Location Prediction

**Figure 6:**
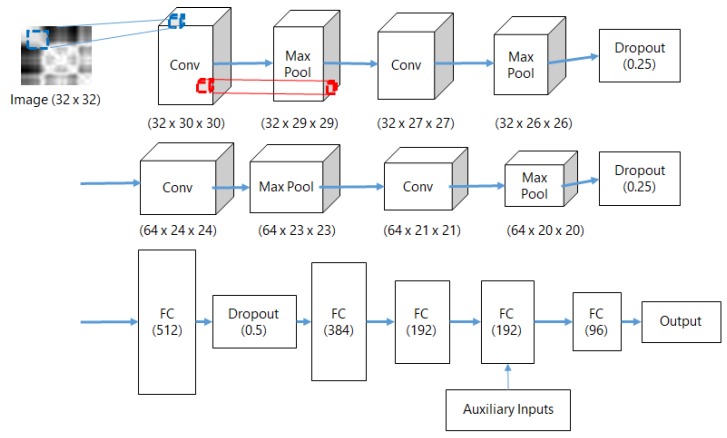
DNN structure.

The structure of a DNN classifier consists of a convolution neural network (CNN) for analyzing image features, and a multi-layer perceptron (MLP) for magnetic landmark classification as described in Figure 6. Every RP is converted to a 32 × 32 size image as an input to the CNN. The CNN structure for analyzing the RP is similar to the CNN structure of the simplified CIFAR-10 dataset classifier. We use two sets of layers that consist of two-dimensional convolution filters, activation function, max pooling layer, and dropout units. Because we append the MLP at the end of the CNN, the final fully connected layer is removed. The MLP receives inputs of size 256 from the CNN and three auxiliary features of size 5. We designed the MLP as cascading fully connected layers that starts from an FC layer of size 196, because we detected up to 99 magnetic landmarks in our test environment. The final layer of the MLP has a size that corresponds to the number of magnetic landmarks in the environment. The DNN classification model is trained with backpropagation. To predict landmarks, features are extracted from classification data and fed to the trained model. The locations are estimated from the locations of predicted landmarks.

## 7. Evaluation

In this section, we describe the experiment setup, and the test environments. Then, we evaluate our system by focusing on three questions: (1) Is it possible to classify magnetic landmarks with the proposed features? (2) What is the overall positioning performance? and (3) Which factor affects the proposed positioning accuracy?

### 7.1. Experiment Setup

**Figure 7:**
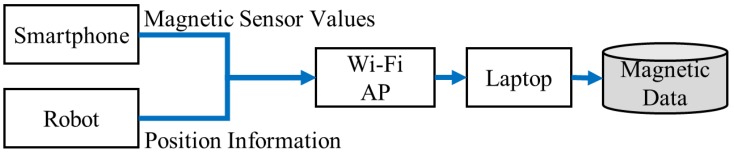
Magnetic data collecting system.

A magnetic data collecting system is implemented to generate classification data for training. The design of the system is shown in Figure 7. We use KOBUKI, an ope-source robot [20]. The robot is controlled with a laptop (Asus TP200S, ASUS, Taipei, Taiwan) that runs ROS Indigo on Ubuntu 14.04. To collect magnetic sensor data, we use a smartphone (Nexus 6P, Huawei, Shenzhen, China) that runs customized ROS Android application. This application reads magnetic data at 100Hz and transmits the data to the laptop through a Wi-Fi connection using a mobile AP. In order to localize the robot, LIDAR-based SLAM is used: Google Cartographer [21], and Gmapping and AMCL from the ROS package. We use a URG radar (Hokuyo URG-04LX, HOKUYO, Osaka, Japan) that can detect the range from 20 mm to 5600 mm. For the test data, we installed magnetic data collection application on a smartphone (Nexus 6P).

### 7.2. Test Environments

**Figure 8:**
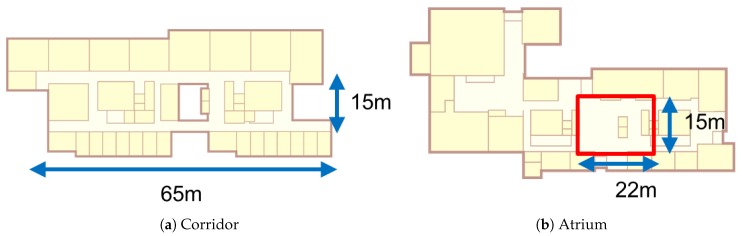
Test area on floor plans.

Experiments were conducted on both a corridor and an atrium, which represent as one-dimensional environment and a two-dimensional environment. The corridor and the atrium have sizes 15 m × 65 m, and 15 m × 22 m as depicted in Figure 8. The overall test environments of these places are listed on Table 1.

Depending on the element of magnetic data used, the locations and the number of detected magnetic landmarks change. Hence, positioning performance may vary depending on which magnetic element is used for positioning. For that reason, we conduct experiments on all magnetic data elements.

The most important condition to control is the consistency of magnetic sensor readings. For collecting the training and testing data set, the same smartphone was used. Before data collection, the smartphone magnetic sensor was calibrated by rotating it on three axes. In addition, changes in the magnetic environment of the test area should be considered. The data collection was performed over three days, and there were no significant changes in the locations of the ferromagnetic objects.

### 7.3. Magnetic Landmark Classification Accuracy

To train the classification model, we used the magnetic data that were used for the magnetic map construction step. However, the trajectory did not cover all test paths. Therefore, we collected additional classification data. For the test path, we designed four different test paths for the corridor and three test paths for the atrium. To test the robustness of the proposed positioning method, each test path did not match a single train path. All training data and test data were collected using the robot to discover ground truth. We collected 2.4 km of training data and 389 m of test data on the corridor. Likewise, 2.3 km of training data and 489 m of test data were collected on the atrium. To train the DNN, we used an Nvidia Titan X graphics card. Approximately 15 min were required for 1500 epochs of training. The overall classification performance was 88.24%. The classification results of all magnetic elements are listed in Table 2.

The classification results of each magnetic element are comprised of three phases. For the first phase, we train the model based only on a single RP image of each magnetic data element. The second phase extends the number of features by using auxiliary inputs. For the final phase, we use all RP images from all magnetic elements.

The classification accuracy in a one-dimensional space was almost perfect even in the first phase. On thecontrary, classification results on a two-dimensional space showed progress with every phase. This experimental observation implies that as the complexity of classification increases, the number of features required for the classification also increases. Moreover, when it comes to the efforts for training the models, the training time increases as the number of classification features decreases. It was notable that training the model for the corridor showed dramatic increases in the number of training epochs. To fully train the model, it took 2000 epochs in the phase 3, 5000 epochs in phase 2, and 7500 epochs in the phase 1.

### 7.4. Magnetic Positioning Performance

Based on the classification accuracy results, we chose the best classification model for each test environment. In both environments, we chose the classification model that detects $m_{z}$ magnetic landmarks for location estimation. To obtain indoor positioning error, we calculated Euclidean distance errors between the locations of users and the locations of the predicted magnetic landmarks.

**Figure 9:**
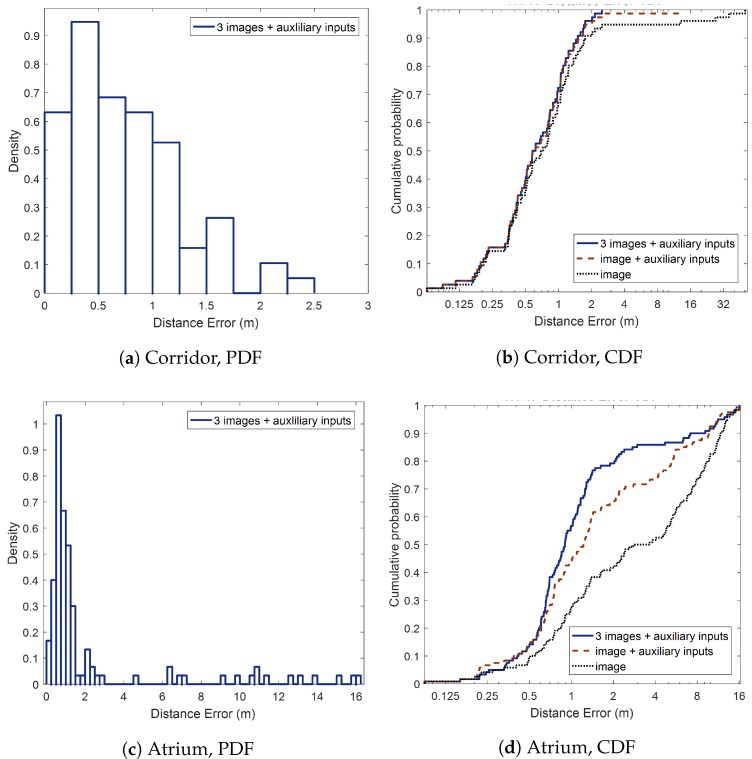
Indoor positioning errors on test environments.

The overall positioning performance is summarized in Table 3. The average positioning error of the corridor (0.76 m) was superior to that of the atrium (2.30 m) because of the significant classification performance difference. For the same reason, from the cumulative distribution functions (CDF) on Figure 9b,d, we observed positioning accuracy enhancement as the number of features increased. The distance between ground truth and corrected magnetic landmarks was 0.8 m on average. Therefore, most of distance errors were located within 2 m, as shown in probability density functions (PDF) on Figure 9a,c. On the contrary, the distance interval between landmarks prediction was 4.52 m for the corridor, and 4.13 m for the atrium. Because people usually walk at the speed of 1.4 m/s [22], the system estimates locations every 3 to 4 s.

### 7.5. Magnetic Map Resolution

The resolution of the magnetic grid map usually affects indoor positioning performance. However, many studies have failed to reveal the relation between map resolution and positioning accuracy. To clarify the relation, we detected magnetic landmarks from different resolution maps, and trained a classification model. We generated three magnetic maps using three different data intervals (0.3 m, 0.6 m, and 0.9 m) using the existing magnetic maps. The positioning results for different magnetic map resolutions are listed in Table 4.

As the resolution decreases, the number of magnetic landmarks also decreases, which confines the number of possible location estimates. Furthermore, low-resolution maps cannot determine the exact locations of magnetic landmarks. For these reasons, the positioning performance degrades as the resolution decreases. However, in the atrium, the positioning error of the 0.3 m resolution map was worse than that of 0.6 m resolution map. This is because, some magnetic landmarks are located too close together on the resolution map, so some classification data were labeled with wrong magnetic landmarks, which degrades the quality of the training data. From the test results, we concluded that at a resolution of least 0.6 m is required to ensure normal positioning performance.

## 8. Conclusions

This paper proposed AMID, an indoor positioning system that estimates locations by recognizing magnetic sequence patterns using a DNN. To our knowledge, this is the first attempt to apply a deep learning technique to a positioning system using only magnetic fingerprints. New features were proposed so that AMID could be used not only for one-dimensional but also for two-dimensional positioning. With the help of a well-trained classifier and a DNN, AMID achieved an accuracy of 0.8 m in a corridor and 2.3 m in an atrium. The results indicate that magnetic positioning is feasible using smartphone sensors alone.

When the performance of AMID was compared with that of the existing methods, AMID showed relatively good positioning results for both accuracy and precision as summarized in Table 5. The test sizes of the methods are also specified in the last column of the table for fair comparison. MaLoc achieved a 1.0 m positioning accuracy, but these results were obtained only in a one-dimensional space (24 m). The performance of AMID in a one-dimensional space was obtained in 15 m × 65 m sized corridors that had a total length in the one-dimensional space of 190 m. This means that AMID is not inferior to MaLoc in accuracy.

Although we focused on the magnetic data for indoor positioning in this study, other signals and sensors can be used together to achieve better positioning accuracy. Sensor fusion, especially with Wi-Fi-based positioning, would make the system more accurate and reliable in the future.

## Figures and Tables

**Table 1:** Test environments.

Place	Magnetic Element	Mean	Standard Deviation	Magnetic Landmarks
Corridor	$m_{xyz}$	50.34 μT	16.11	88
	$m_{xy}$	34.11 μT	15.88	94
	$m_{z}$	−33.86 μT	14.46	99
Atrium	$m_{xyz}$	46.02 μT	10.76	56
	$m_{xy}$	25.83 μT	11.05	64
	$m_{z}$	−35.68 μT	12.42	43

**Table 2:** Magnetic classification accuracy.

Place	Magnetic Element	Training Data	Test Data	Phase 1	Phase 2	Phase 3
Corridor	$m_{xyz}$	1240	77	97.4%	98.7%	100%
	$m_{xy}$	1288	76	93.4%	98.7%	100%
	$m_{z}$	1322	78	97.4%	100%	100%
Atrium	$m_{xyz}$	1421	122	36.1%	45.1%	59.0%
	$m_{xy}$	1426	130	30.8%	47.7%	67.7%
	$m_{z}$	1265	120	41.7%	68.3%	80.8%

**Table 3:** Magnetic indoor positioning results.

Place		Corridor	Atrium
Accuracy	Mean	0.76 m	2.30 m
Precision	Within 90%	1.50 m	8.14 m
	Within 50%	0.60 m	0.90 m

**Table 4:** Indoor positioning results on different map resolution.

Place	Map Resolution	Magnetic Landmarks	Classification Accuracy	Positioning Error
Corridor	0.3	88	100%	0.82 m
	0.6	52	100%	1.18 m
	0.9	24	100%	2.77 m
Atrium	0.3	43	80.8%	2.30 m
	0.6	30	79.7%	2.08 m
	0.9	18	82.8%	2.59 m

**Table 5:** Indoor positioning performance comparison.

System	Positioning Method	Accuracy	Precision	Test Size
AMID	Deep Leaning-based Classification	1.7 m	90% within 5.6 m 50% within 0.8 m	22 m × 15 m 15 m × 65 m
MaLoc [6]	Particle Filter	1.0 m	80% within 1.8 m 50% within 0.7 m	72 m × 64 m 24 m
Chung et al. [4]	RMS Error	4.7 m	90% within 5.9 m 50% within 2.5 m	15 m × 20 m 187 m
LocateMe [12]	DTW	3.4 m	90% match	38 m 51 m
